# Antibiotic Resistance in Dentistry: A Review

**DOI:** 10.3390/antibiotics14121259

**Published:** 2025-12-12

**Authors:** Mohammed Zahedul Islam Nizami, Iris Xiaoxue Yin, John Yun Niu, Ollie Yiru Yu, Chun Hung Chu

**Affiliations:** 1 Department of Mineralized Tissue Biology, ADA Forsyth Institute, 100 Chestnut Street, Somerville, MA 02143, USA; 2 Faculty of Dentistry, The University of Hong Kong, Hong Kong SAR, China; irisxyin@hku.hk (I.X.Y.); niuyun1@connect.hku.hk (J.Y.N.); ollieyu@hku.hk (O.Y.Y.)

**Keywords:** antibiotics, dentistry, prevention, oral health, drug resistance, public health

## Abstract

**Background:** The widespread use of antibiotics in dentistry has become a significant driver of the global rise in antibiotic resistance, posing serious risks to both oral and overall health. **Objectives:** This study aims to review antibiotic use in dentistry, elucidates the mechanisms of resistance development, identifies contributing factors, and discusses strategies to mitigate this growing global health threat. **Methods:** This narrative review examines current patterns of antibiotic prescribing in dentistry and evaluates evidence showing that antibiotics, although essential for preventing and managing odontogenic infections, are often prescribed unnecessarily or inappropriately. **Results:** The analysis highlights the growing resistance of key oral pathogens such as *Streptococcus* spp., *Enterococcus faecalis*, and *Porphyromonas gingivalis*, which increasingly limits the effectiveness of conventional treatments. Factors contributing to this trend include inconsistent adherence to clinical guidelines, patient pressure, and insufficient awareness of antibiotics stewardship among dental professionals. To address these challenges, the review emphasizes the importance of evidence-based prescription, strengthened stewardship programs, and the development of alternative therapies, including host-modulating agents and bacteriophage applications. Ongoing education and professional development are equally vital to enhance clinical judgment and promote responsible prescribing habits. **Conclusions:** Overcoming antibiotic resistance in dentistry requires coordinated effort among clinicians, researchers, educators, and policymakers. Expanding surveillance, enforcing stewardship-driven policies, and supporting innovation in therapeutic research are key to reducing antibiotic misuse and preserving their effectiveness. Through collective commitment and informed practice, the dental profession can play a crucial role in protecting antibiotic efficacy and promoting sustainable, high-quality patient care.

## 1. Introduction

The discovery and widespread use of antibiotics in the early 20th century marked a turning point in medical history, revolutionizing the treatment of infectious diseases and significantly reducing global mortality rates. The discovery of penicillin in 1928 and its subsequent mass production ushered in a new era of antibiotics therapy [1,2]. Over the past several decades, the development of broad-spectrum antibiotics has transformed healthcare by enabling providers to effectively treat bacterial infections that once caused severe illness and death. These agents have become essential not only in general medicine but also in dentistry, where infections of odontogenic origin are common and can lead to serious systemic complications if not properly managed [3,4,5,6,7].

In dental practice, antibiotics are routinely prescribed to manage acute and chronic infections, support periodontal therapy, and provide prophylaxis before invasive procedures. They help limit bacterial proliferation, reduce inflammation, and prevent the systemic spread of oral pathogens. It is important to recognise that inappropriate antibiotic prescription is a complex, multi-faceted challenge driven by healthcare providers across all disciplines. Within this broader context, however, dentistry represents a significant and measurable contributor; estimates suggest that dental prescriptions account for approximately 10% of all antibiotic prescriptions in primary healthcare settings [8]. Despite their unquestionable benefits, the overuse and misuse of antibiotics have raised serious concerns due to the escalating threat of antibiotic resistance [9,10].

Antibiotic resistance occurs when bacteria develop mechanisms to survive exposure to antibiotics, rendering these drugs ineffective. Resistance typically arises through genetic mutations or horizontal gene transfer, giving rise to strains that can withstand standard treatments [11,12]. The impact is serious: infections become increasingly difficult to treat, often necessitating the use of stronger or more toxic drugs, prolonged hospitalization, or surgical intervention. The World Health Organization (WHO) has warned that without urgent global action, we may enter a post-antibiotic era, where even minor infections could once again become fatal [13]. In dentistry, the inappropriate and excessive use of antibiotics has significantly contributed to the emergence of resistant bacteria [14]. Contributing factors include diagnostic uncertainty, patient expectations for rapid relief, limited adherence to clinical guidelines, and reliance on empirical prescribing. Moreover, the oral cavity harbors a complex microbiome containing pathogenic species capable of acquiring and disseminating resistance genes through various mechanisms [15,16]. The rise of resistant oral pathogens complicates routine dental procedures and periodontal therapy, leading to treatment failures and increased dependence on broad-spectrum or more potent antibiotics.

A critical and often overlooked factor is that dental clinics can serve as reservoirs for antibiotic-resistant bacteria such as methicillin-resistant *Staphylococcus aureus*, facilitating their nosocomial transmission via contaminated surfaces and colonization of healthcare personnel [17]. This underscores the urgent need for antibiotic stewardship within dental practice. Efforts to optimize antibiotic use, enhance diagnostic accuracy, and raise awareness among practitioners and patients are crucial to counter the progression of antibiotic resistance [10]. Addressing antibiotic resistance in dental settings is vital not only to preserve the efficacy of current antibiotics but also to safeguard public health and ensure the continued success of infection management in dental care. This review aims to provide a comprehensive overview of antibiotic use in dentistry, elucidate the mechanisms of resistance development, identify contributing factors, and discuss evidence-based strategies to mitigate this growing global health threat.

## 2. Antibiotic Use in Dentistry

In dental practice, antibiotics are essential for managing both odontogenic and non-odontogenic infections and for preventing systemic complications associated with oral diseases. When prescribed appropriately, they can markedly improve patient outcomes. However, inappropriate, excessive, or delayed antibiotic prescribing continues to contribute substantially to the development of antibiotic resistance within the oral microbiome. For instance, studies show that only about 12% of dentists fully adhere to prescribing guidelines, while broader-spectrum agents, such as amoxicillin–clavulanate or clindamycin, are frequently used even when narrower-spectrum alternatives would be sufficient [18]. Deviation from guideline-driven therapy increases selective pressure on oral pathogens and disrupts microbial balance. Ensuring judicious, evidence-based antibiotic use requires a clear understanding of appropriate clinical indications, such as acute spreading infections, systemic involvement, or prophylaxis in high-risk patients along with knowledge of commonly used agents and the practitioner, patient, and specialty factors that influence prescribing behavior [14].

The primary indications for antibiotic use in dentistry include:

*1.*  *Management of odontogenic infections with systemic involvement;*
*2.*  *Treatment of acute periodontal and periapical abscesses when operative care alone is insufficient;*
*3.*  *Infective endocarditis prophylaxis for patients at the highest risk;*
*4.*  *Post-operative infection prevention in selected surgical cases.*

Importantly, antibiotics should not be used for inflammatory conditions such as symptomatic irreversible pulpitis, as they offer no clinical benefit and contribute to unnecessary antibiotic exposure [19].

### 2.1. Odontogenic Infections

Odontogenic infections, including periapical abscesses, periodontal abscesses, and cellulitis, are most effectively managed with local interventions such as drainage, pulpotomy, or root canal therapy. Antibiotics are only indicated when systemic signs are present, including fever, malaise, trismus, diffuse swelling, lymphadenopathy, or when the patient is immunocompromised [18]. Despite this, studies continue to report unnecessary prescriptions in situations where operative management alone would be effective.

### 2.2. Periodontal Diseases

Periodontal diseases, including gingivitis and periodontitis, are chronic bacterial infections affecting the supporting structures of the teeth. In specific cases, particularly aggressive, refractory, or advanced (stage III/IV) periodontitis, adjunctive systemic antibiotics such as amoxicillin combined with metronidazole may be indicated when mechanical therapy alone is insufficient, targeting pathogens like *Actinobacillus actinomycetemcomitans*, *Porphyromonas gingivalis*. Clinical evidence shows that this combination can yield greater improvements in probing depth and attachment levels compared with scaling and root planning alone [20]. However, routine antibiotic use in mild or chronic cases is discouraged due to limited benefits and the risks of antibiotic resistance and adverse effects [21,22].

### 2.3. Prophylaxis

Antibiotic prophylaxis is reserved for patients at the highest risk of infective endocarditis, immunocompromised individuals, and those undergoing invasive surgical procedures under specific conditions. Current guidelines, including those from the American Heart Association, recommend targeted regimens for high-risk patients undergoing invasive dental procedures, highlighting the need for evidence-based, rather than routine, prophylaxis [23]. Despite this, overuse of prophylactic antibiotics remains common in dentistry, even when not indicated, and recent evidence shows that many surgical procedures, such as extractions, implants, or periodontal surgeries, do not require antibiotics when proper aseptic techniques are followed, underscoring the importance of avoiding unnecessary prescriptions [24,25].

### 2.4. Post-Operative Use

Post-operative antibiotics are often unnecessarily prescribed following extractions, implant placement, or periodontal surgery. Current evidence demonstrates that, with effective infection-control measures and minimally invasive techniques, routine post-operative antibiotic use is seldom justified [26].

### 2.5. Commonly Prescribed Antibiotics

Commonly prescribed antibiotics in dentistry include penicillins such as amoxicillin (the first-line agent for most odontogenic infections), amoxicillin–clavulanate, metronidazole for anaerobic coverage, and clindamycin, which is traditionally used for patients with penicillin allergy. However, recent antibiotics stewardship guidelines discourage the routine use of clindamycin because of its association with *C. difficile* infection and increasing resistance patterns [18,27,28,29]. Despite clear therapeutic indications, antibiotic overprescription remains a persistent challenge in dental practice. Key drivers include patient expectations for rapid pain relief, misconceptions that antibiotics treat dental pain itself, diagnostic uncertainty. They limited familiarity or engagement with evidence-based guidelines, defensive prescribing due to medicolegal concerns, and time constraints in emergency settings [14,30,31]. Furthermore, inadequate management of the underlying dental pathology, such as untreated caries, pulpal disease, or chronic periodontal infection, often leads to reliance on antibiotics instead of definitive interventions, contributing to unnecessary exposure, adverse effects, and the acceleration of antibiotic resistance [30,32,33].

## 3. Mechanisms of Antibiotic Resistance in Oral Pathogens

Antibiotic resistance arises through complex biological processes that enable bacteria to survive exposure to antibiotics designed to inhibit or kill them [34]. These mechanisms are primarily driven by genetic changes, including spontaneous mutations and horizontal gene transfer, which confer survival advantages under selective pressure from antibiotic use. Understanding these mechanisms is essential for developing strategies to combat antibiotics resistance in oral pathogens, including *Streptococcus* spp., *Porphyromonas* spp., *Enterococcus faecalis* [35,36,37].

### 3.1. Resistance Actions

Antibiotics resistance in oral pathogens is conferred through specific biochemical mechanisms of action that render antibiotics ineffective including enzymatic drug inactivation, target modification, efflux pumps, and reduced membrane permeability.

***Enzymatic Degradation and Modification:*** Some bacteria produce enzymes that inactivate antibiotics before they reach their targets [38,39]. For example, β-lactamases produced by *Streptococcus* spp. and *Enterococcus faecalis* hydrolyze β-lactam antibiotics [39,40], and β-lactamase activity in *Porphyromonas* spp. reduces susceptibility to penicillins commonly used in dental therapy [40,41].

***Target Modification:*** Bacteria can alter antibiotic-binding sites, thereby decreasing drug affinity [42,43]. For instance, *E. faecalis* modifies penicillin-binding proteins (PBPs), reducing β-lactam efficacy [42,43]; macrolide resistance in *Streptococcus* spp. occurs via ribosomal methylation [44,45]; and *P. gingivalis* exhibits structural changes in lipopolysaccharides or outer membrane components, which lower drug binding and penetration [43].

***Efflux Pumps:*** Efflux systems expel antibiotics from bacterial cells, reducing intracellular concentrations and contributing to resistance [44,45]. For example, efflux pumps in *Streptococcus* spp. (e.g., mef, mel) confer macrolide resistance, while efflux systems in *Porphyromonas gingivalis* reduce susceptibility to tetracyclines and other agents, often leading to multidrug resistance [44,45].

***Reduced Permeability:*** Altered membrane permeability in Gram-negative anaerobes limits antibiotic entry, contributing to treatment failure in periodontal infections [46,47].

### 3.2. Mechanisms of Resistance Acquisition

The genetic determinants for resistant actions are established and spread gene acquisition via horizontal gene transfer and spontaneous mutation.

***Horizontal Gene Transfer:*** Horizontal gene transfer allows bacteria to acquire resistance genes from other microorganisms [48,49]. This can occur through conjugation, which involves plasmid transfer via cell–cell contact; transduction, mediated by bacteriophages; or transformation, which involves uptake of free DNA from the environment. HGT facilitates the spread of β-lactamase genes, macrolide resistance determinants, and efflux pump genes, particularly within plaque biofilms [48,49].

***Spontaneous Mutation:*** Chromosomal mutations can alter antibiotic targets, upregulate efflux systems, or modify metabolic pathways, contributing to resistance [34,36]. Examples include fluoroquinolone target site mutations, PBP mutations in *E. faecalis* [42,43], and metronidazole resistance in *Porphyromonas gingivalis* through metabolic gene alterations [50].

### 3.3. Clinically Relevant Oral Pathogens

Clinically relevant oral pathogens exhibit diverse mechanisms of antibiotic resistance. *Streptococcus* spp. shows β-lactam resistance through PBP modifications and macrolide resistance via efflux systems and target methylation [51]. *Enterococcus faecalis* demonstrates β-lactamase production, PBP mutations, and multidrug resistance, particularly in persistent endodontic infections [52]. *Porphyromonas gingivalis* exhibits reduced membrane permeability, enzymatic inactivation, and altered outer-membrane structures, with rising metronidazole resistance complicating periodontal therapy [50].

**Figure 1:**
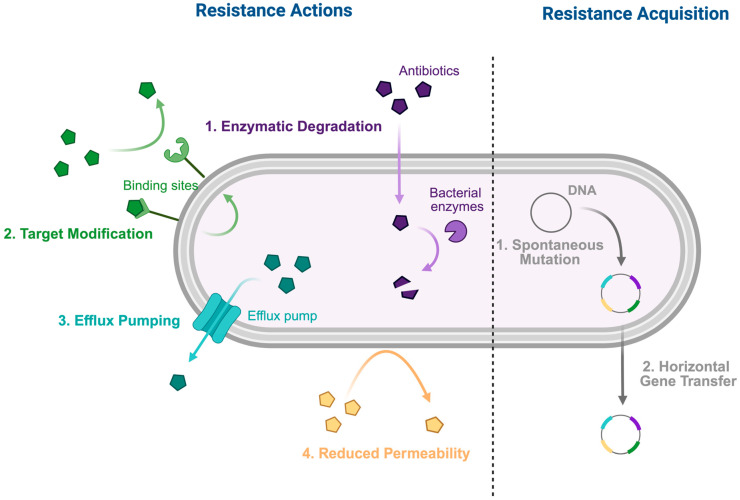
Mechanisms of Antibiotic Resistance.

In summary, antibiotics resistance arises and spreads through diverse and dynamic mechanisms. Figure 1 summarizes the mechanisms of antibiotic resistance. Oral pathogens’ capacity to acquire and share resistance genes intensifies the challenge of infection management, highlighting the need for rational antibiotic use, continuous surveillance, and innovative research. Effective antibiotics stewardship in dentistry is essential to preserve antibiotic efficacy and safeguard public health for future generations [53].

***Resistance Actions:*** 

- *Enzymatic degradation or modification: Bacterial enzymes chemically modify or break down antibiotics.*
- *Target modification: The binding sites of antibiotics alter molecular structures.*
- *Efflux pumps: Bacteria expel antibiotics from cytoplasm through efflux pumps.*
- *Reduced membrane permeability: Loss of porin channels in the membrane decreases drug uptake.*

***Resistance Acquisition:*** 

- *Spontaneous mutations: Resistance genes arise from random chromosomal mutations.*
- *Horizontal gene transfer: Resistance genes are transferred from one bacterium to another.*

## 4. Factors Contributing to Resistance in Dentistry

The escalation of antibiotic resistance in dental practice arises from multiple interconnected factors, creating significant challenges for effective infection management. A primary contributor is inappropriate prescribing practices. Dentists, often under pressure to provide rapid solutions or meet patient expectations, may prescribe antibiotics unnecessarily or select broad-spectrum agents when narrower alternatives would suffice. Empirical prescribing without a clear clinical indication fosters selective pressure on bacteria, promoting the emergence and proliferation of resistant strains. This issue is compounded by the limited availability of definitive diagnostic tools in many dental settings, leading practitioners to rely on clinical judgment rather than microbiological evidence. Without precise identification of the causative pathogens, antibiotics may be ineffective against the target bacteria, inadvertently encouraging resistance [54,55,56,57].

Poor guideline adherence and limited awareness and knowledge among some practitioners regarding current guidelines and antibiotics stewardship principles is another key factor. Despite growing evidence advocating judicious antibiotic use, many clinicians lack updated training or familiarity with the latest recommendations [32]. This knowledge gap results in inconsistent prescribing behaviors, with outdated practices persisting or antibiotics being overused in cases where local measures or surgical interventions suffice [58]. While continuing education and professional development are crucial to bridging this gap, access to such resources remains uneven across regions and practices [59].

Patient demands also drive unnecessary antibiotic use. Many patients perceive antibiotics as a quick solution for dental pain or swelling [60]. Dentists, aiming to meet patient expectations or avoid dissatisfaction, may prescribe antibiotics even when not indicated [61]. Misconceptions about antibiotic efficacy and safety further reinforce a culture where antibiotics are treated as routine rather than targeted therapy [30,62].

Limited diagnostic capabilities exacerbate these challenges. In numerous dental clinics, rapid microbiological testing is unavailable, forcing reliance on clinical signs and symptoms alone [63,64]. This empirical approach increases the likelihood of inappropriate prescriptions and hampers the ability to distinguish bacterial from non-bacterial conditions or to identify the specific pathogens involved [65,66].

Infection control and sterilization practices are critical in preventing the spread of resistant bacteria within dental settings [67]. Inadequate sterilization, cross-contamination, and poor infection control protocols can create reservoirs of resistant strains among patients and staff, complicating future infection management and contributing to community-level spread [68].

The limited stewardship awareness dental clinic itself can become a node for the transmission of resistant strains. Evidence confirms the nosocomial (healthcare-associated) transmission of antibiotic-resistant bacteria within dental settings. Resistant pathogens can persist on environmental surfaces, dental unit waterlines, and inadequately sterilized instruments, creating a reservoir for cross-contamination. Studies reported the presence of resistant bacteria in dental offices and even the colonization of dental healthcare workers, highlighting a transmission cycle that can perpetuate resistance independent of prescribing practices [69]. This environmental dimension underscores that infection control is not only about preventing acute infections but is a fundamental pillar of comprehensive antibiotics stewardship.

The consequences of these factors are profound. Resistant infections tend to be more persistent and difficult to treat, often requiring more potent, toxic, or combination therapies, which increase side effects and healthcare costs. For instance, inappropriate dental antibiotic prescriptions for infective endocarditis prophylaxis in the U.S. are estimated to result in excess costs of approximately US$31 million annually, including adverse events and out-of-pocket expenses [70]. More broadly, antibiotic-resistant infections in inpatient settings have been associated with incremental costs exceeding US$1300 per case compared with non-resistant infections [71]. Resistance also undermines empiric therapy, prompting clinicians to use broader-spectrum or last-line agents, which accelerates further resistance development.

## 5. Strategies to Combat Antibiotic Resistance in Dentistry

Combating antibiotic resistance in dentistry requires a comprehensive, multifaceted approach that addresses the factors driving inappropriate antibiotic use. The primary and most effective strategy is to prevent infections, thereby reducing the need for antibiotic therapy. When antibiotics are genuinely indicated, a parallel strategy of robust antibiotics stewardship must ensure their judicious, evidence-based use [72].

### 5.1. Prevention as a Primary Strategy to Reduce Antibiotic Demand

The cornerstone of mitigating antibiotic resistance is to minimize the incidence of infections that might require antibiotics treatment. This involves proactive measures at the patient, procedural, and environmental levels.

***Patient-Centered Oral Hygiene and Education:*** Effective daily mechanical plaque control through toothbrushing and interdental cleaning (e.g., floss, interdental brushes) is fundamental in preventing caries and periodontal disease, which are the primary sources of odontogenic infections. Dental professionals play a critical role in providing individualized oral hygiene instruction and motivational counseling. Public health campaigns and regular dental check-ups reinforce these practices, enabling early detection and management of issues before they progress to acute infections [73,74].

***Early and Minimally Invasive Treatment of Oral Diseases:*** Prompt intervention for early-stage dental diseases prevents progression to acute, systemic infections. The early restoration of carious lesions, timely pulpal therapy (e.g., pulp capping, root canal treatment), and management of incipient periodontal disease through debridement can resolve pathology locally. These definitive treatments eliminate the source of infection, rendering systemic antibiotics unnecessary for localized conditions, and underscore the principle that the primary treatment for most odontogenic infections is mechanical or surgical intervention [75,76].

***Rigorous Infection Prevention and Control in Dental Practice:*** Rigorous clinical infection prevention and control protocols are essential to prevent postoperative and surgical site infections, thereby obviating the need for prophylactic or therapeutic antibiotics. The use of antiseptic mouth rinses (e.g., chlorhexidine gluconate) prior to surgical procedures reduces the oral microbial load [77]. Employing a rubber dam during endodontic treatment isolates the operating field, minimizing contamination from oral flora and enhancing treatment success [78]. Meticulous soft tissue handling and proper wound management are critical. Adherence to strict protocols for sterilizing reusable instruments and disinfecting clinical surfaces is vital to prevent cross-contamination and the spread of resistant pathogens within the dental setting [67,69]. Effective IPC renders routine postoperative antibiotic prescriptions redundant for most dentoalveolar surgeries.

### 5.2. Antibiotics Stewardship to Ensure Judicious Use

When antibiotic therapy is indicated, its use must be optimized through structured stewardship programs and adherence to guidelines to minimize selective pressure and preserve efficacy. Robust antibiotics stewardship programs promote judicious use through standardized protocols that emphasize selecting the most appropriate antibiotic, determining the correct dose, and limiting therapy to the shortest effective duration Adherence to these principles reduces unnecessary antibiotic exposure, thereby decreasing the selective pressure that drives resistance.

***Evidence-Based Clinical Guidelines:*** Equally important is strict adherence to evidence-based clinical guidelines from authoritative organizations, such as the American Dental Association (ADA) and the WHO [79,80]. Following these recommendations promotes consistency in clinical practice and ensures that antibiotic use aligns with current scientific understanding, minimizing overprescription and the risk of resistance.

***Professional Education:*** Education and ongoing training for dental professionals are crucial for enhancing awareness of antibiotics stewardship principles. Greater awareness of the consequences of antibiotic resistance fosters a culture of stewardship within the dental community. Additionally, educating clinicians about alternative management strategies, such as local measures, surgical interventions, or watchful waiting, can reduce reliance on antibiotics and encourage more conservative, evidence-based approaches [81,82].

***Advanced Diagnostic Technologies:*** Advances in diagnostic technologies offer another critical tool in combating antibiotic resistance. Point-of-care diagnostic tests can provide timely and accurate identification of bacterial pathogens and resistance markers, enabling clinicians to prescribe targeted therapy rather than relying on broad-spectrum empirical treatment [54,83,84].

***Patients and the Public’s Education:*** Public awareness and patient education are also essential. Informing patients about the appropriate indications for antibiotics, their futility in managing inflammatory conditions (e.g., pulpitis), and the public health threat of resistance is crucial for aligning expectations and diminishing demands for unwarranted prescriptions [85]. When patients understand the risks of unnecessary antibiotics to themselves, their contacts, and society, requests for inappropriate prescriptions decline significantly [86].

Collectively, these interconnected strategies of prevention and stewardship are mutually reinforcing. Effective implementation requires collaboration among clinicians, researchers, policymakers, and the broader community. By fostering a culture of responsible antibiotic use, the dental profession can make a meaningful contribution to global efforts against antibiotic resistance, ensuring that these vital drugs remain effective for future generations.

## 6. Future Directions

Addressing antibiotic resistance in dentistry and broader healthcare requires innovative research and proactive policy measures. A key priority is the development of alternative antibacterial agents that can effectively target resistant bacteria without promoting further resistance. Promising approaches include antibacterial peptides, phage therapy, and nanomaterials, which can bypass traditional resistance mechanisms [87,88,89]. These strategies offer targeted treatment with fewer side effects and minimal disruption to the normal microbiota, thereby reducing selective pressure for resistance.

Vaccines also represent a promising avenue for preventing bacterial infections that commonly require antibiotic treatment. Developing vaccines against oral pathogens such as *Streptococcus mutans*, *Porphyromonas gingivalis*, or *Enterococcus faecalis* could significantly lower infection rates and reduce the need for antibiotics [90]. By shifting the focus from treatment to prevention, vaccines have the potential to transform infection control in dentistry.

Probiotics provide an additional complementary strategy by promoting a healthy oral microbiome that resists colonization by pathogenic bacteria. Restoring microbial balance may decrease reliance on antibiotics and reduce the risk of resistance. Ongoing research into specific probiotic strains aims to determine their effectiveness in preventing or managing oral infections [91].

Policy measures are equally vital. Governments and health organizations should implement robust surveillance systems to monitor resistance patterns across regions and populations, providing critical data to inform clinical guidelines and support responsible prescribing [92]. Regulating the sale and prescription of antibiotics is essential to prevent over-the-counter misuse and unregulated dispensing, both major contributors to resistance [92]. Educational campaigns targeting healthcare providers and the public can further raise awareness of responsible antibiotic use and foster a culture of stewardship [93].

In summary, a combination of innovative research, effective prevention strategies, and strict policy enforcement will be critical in combating antibiotic resistance. By integrating these approaches, the dental profession and broader healthcare community can help preserve the effectiveness of antibiotics and safeguard public health for future generations.

## 7. Conclusions

Antibiotic resistance in dentistry poses a serious threat to oral and systemic health, compromising the effectiveness of commonly used antibiotics and increasing the risk of prolonged or complicated infections. Mitigating this crisis requires rational, evidence-based antibiotic use, supported by robust stewardship programs, ongoing professional education, and the adoption of alternative management strategies such as local measures and preventive care. Equally important is collaboration among clinicians, researchers, policymakers, and patients to implement stewardship, promote public awareness, and develop innovative therapies. By fostering a culture of responsible antibiotic use, the dental profession can help preserve the efficacy of antibiotics, protect public health, and ensure these vital drugs remain effective for future generations.

## Data Availability

Not applicable.
